# Supplementary material for: Real-time health monitoring through urine metabolomics
Source: NPJ Digit Med. 2019 Nov 11;2:109. doi: 10.1038/s41746-019-0185-y (PMC6848197; doi:10.1038/s41746-019-0185-y)
Supplement: Supplementary file 1 — Supporting Information [file 41746_2019_185_MOESM1_ESM.pdf]

Supporting Information

Real time health monitoring through urine metabolomics

Ian J. Miller[1], Sean R. Peters[2], Katherine A. Overmyer[3], Brett R. Paulson[1,3], Michael S. Westphall[1,4], and Joshua J. Coon[1,2,4]*

[1]National Center for Quantitative Biology of Complex Systems, Madison, WI, [2]Department of Chemistry, University of Wisconsin-Madison, Madison, WI, [3]Morgridge Institute for Research, Madison, WI, [4]Department of Biomolecular Chemistry, University of Wisconsin-Madison, Madison, WI

*Correspondence to:
Joshua J. Coon, Ph.D.
Professor, Departments of Chemistry and Biomolecular Chemistry
University of Wisconsin-Madison
425 Henry Mall, Room 4426
Madison, WI 53706
jcoon@chem.wisc.edu

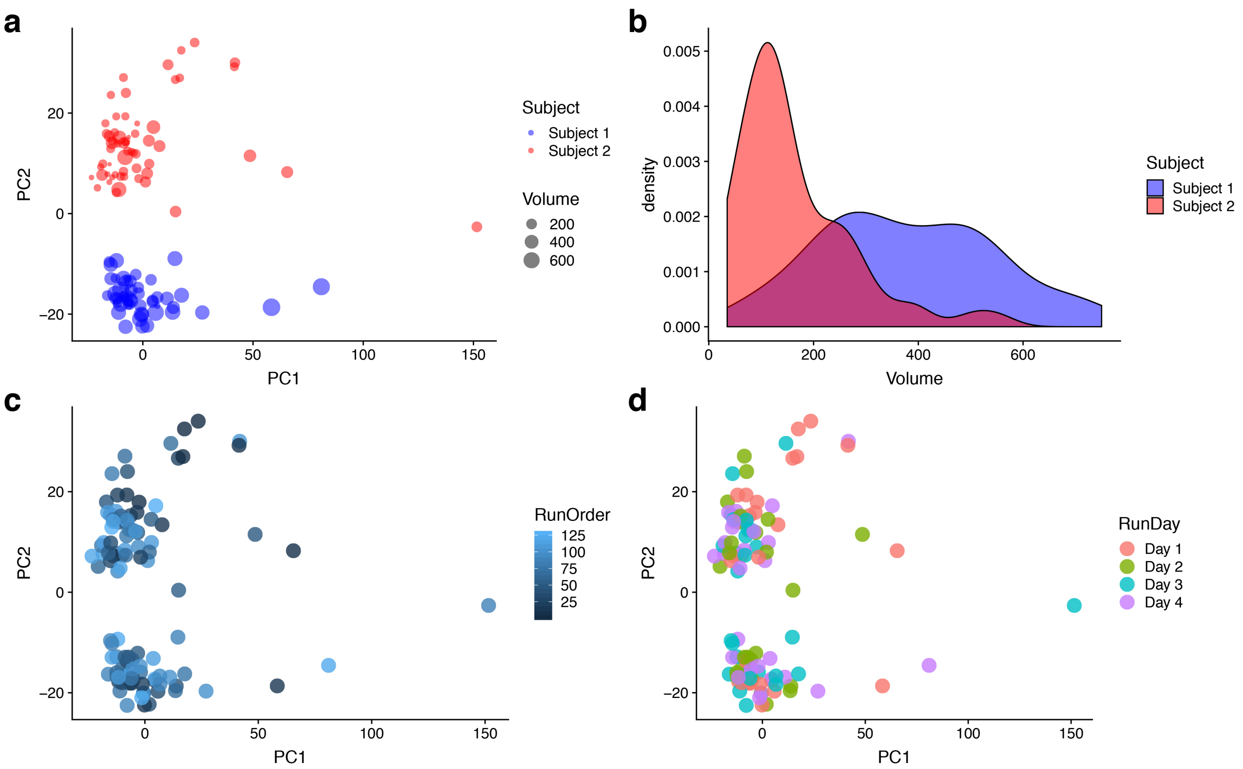

Supplementary Figure 1. PCA-based quality control analysis. (**a**) Samples colored by Subject and sized by volume. (**b**) Density plot of sample volumes for Subject 1 and Subject 2. (**c**) Sample points colored by run order. (**d**) Sample points colored by run day. See **Supplementary Dataset 3** for quantitative values.

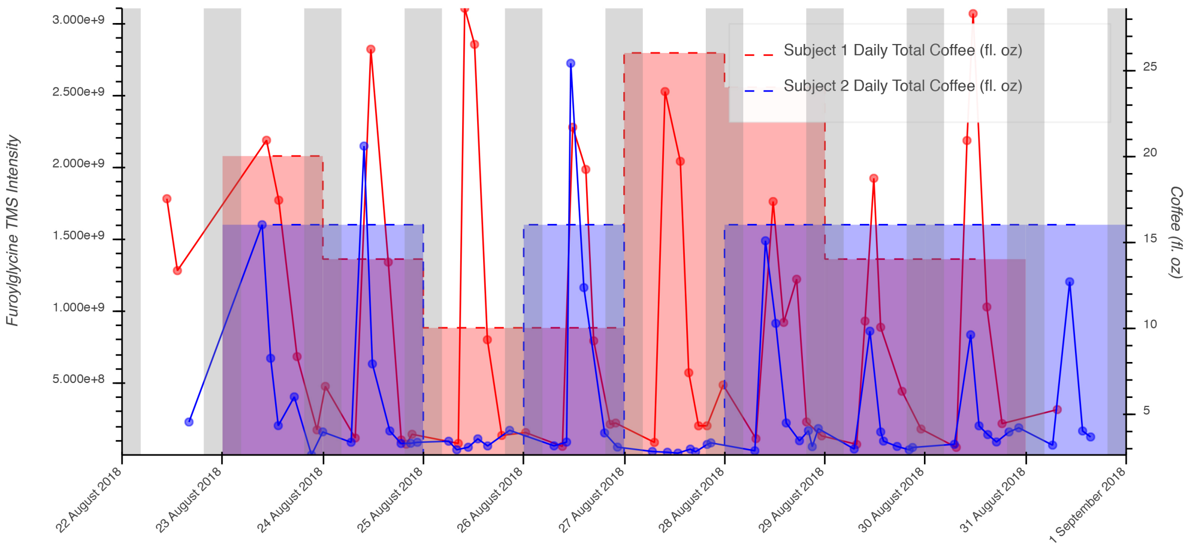

Supplementary Figure 2. Intensity of furoylglycine TMS over time for both subjects. Coffee (fl. oz) vs. log2-furoylglycine TMS (daily average intensity); repeated measures r = 0.617, p = 0.011, q = 0.201, dof = 14.

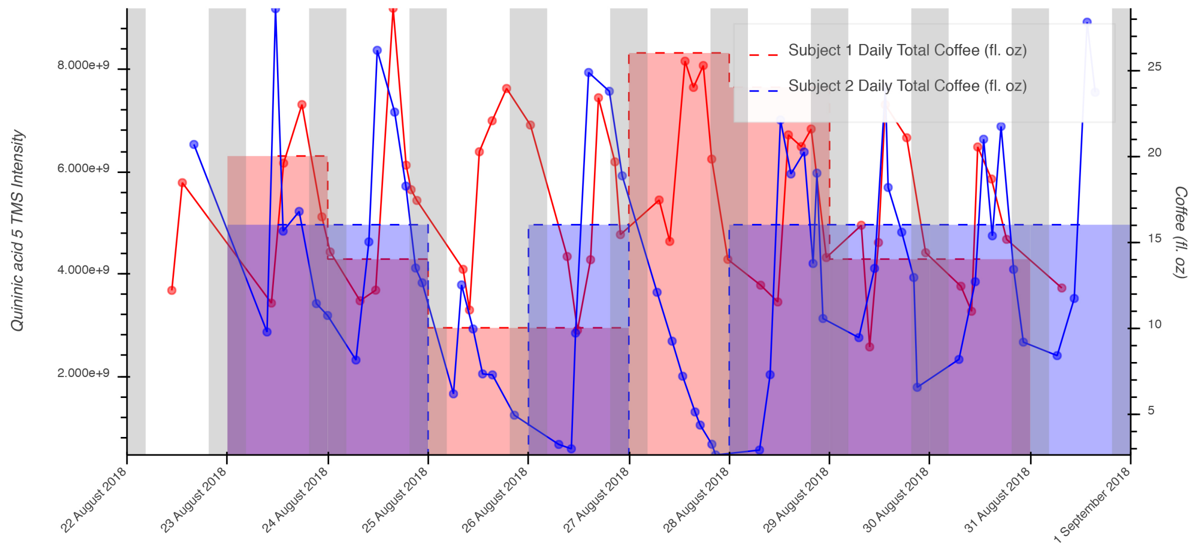

Supplementary Figure 3. Intensity of quininic acid 5 TMS over time for both subjects. Coffee (fl. oz) vs. log2-quininic acid 5 TMS (daily average intensity); repeated measures r = 0.787, *p* = 2.93e-4, *q* = 0.0884, dof = 14.

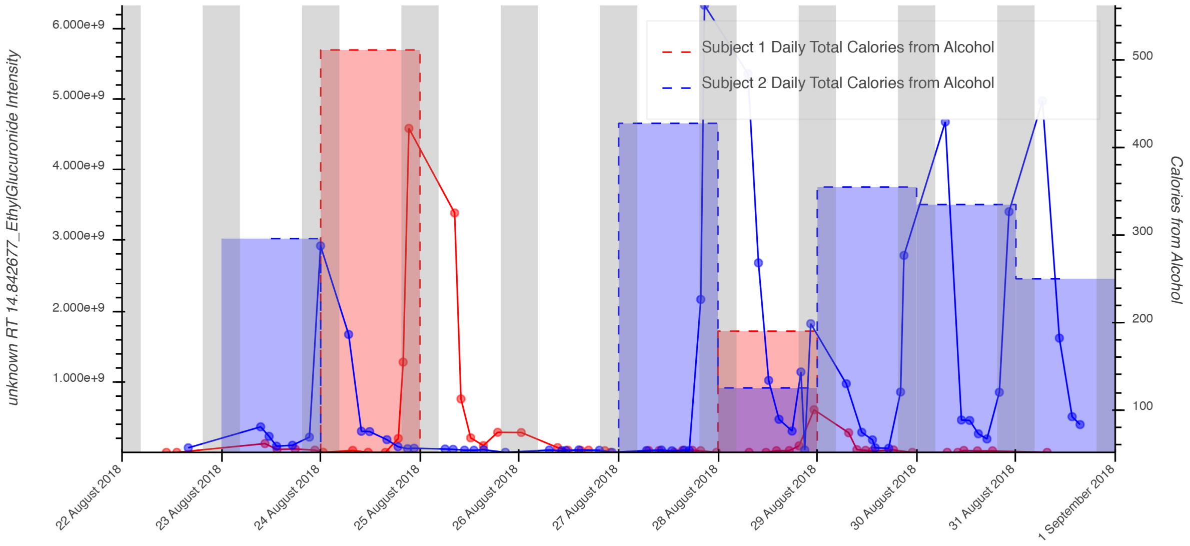

Supplementary Figure 4. Intensity of ethyl glucuronide over time for both subjects. Calories from Alcohol vs. log2-ethyl glucuronide (daily average intensity); repeated measures r = 0.657, *p* = 0.006, *q* = 0.0508, dof = 14.

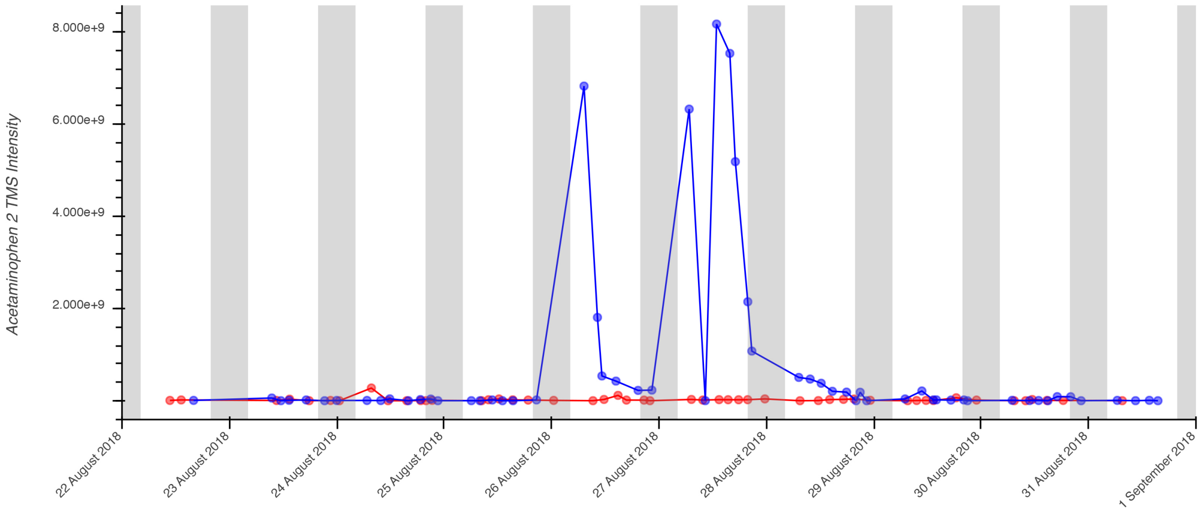

Supplementary Figure 5. Intensity of acetaminophen 2 TMS over time for both subjects. No quantitative data were recorded for acetaminophen consumption, so correlation metrics are not available.

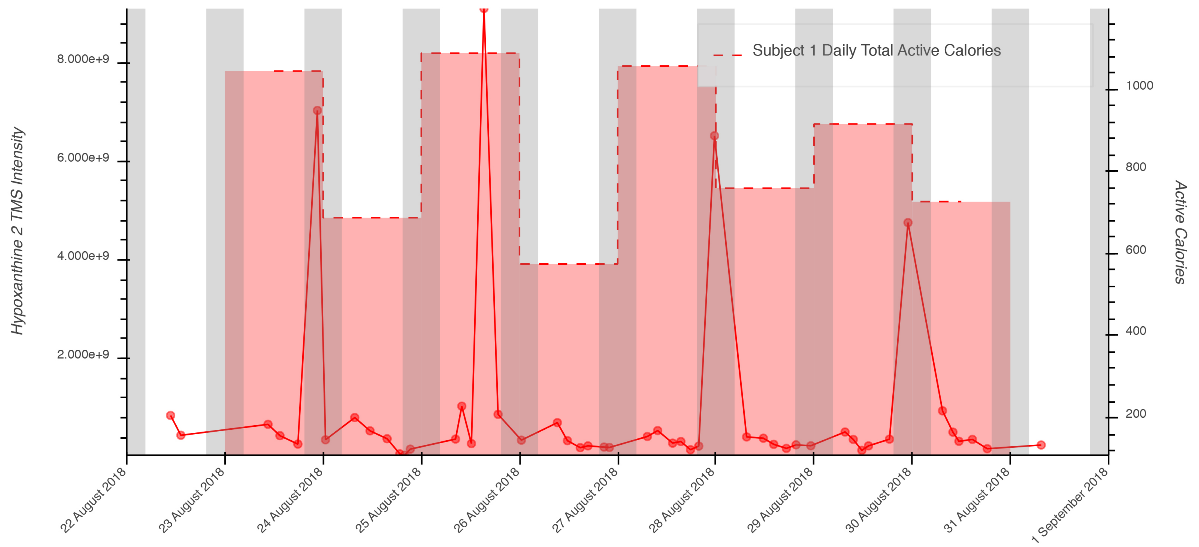

Supplementary Figure 6. Intensity of hypoxanthine 2 TMS over time for both subjects. Active Calories vs. log2-hypoxanthine 2 TMS (daily average intensity); Spearman's Rho: 0.833, p = 0.0102, q = 0.472, n = 8.

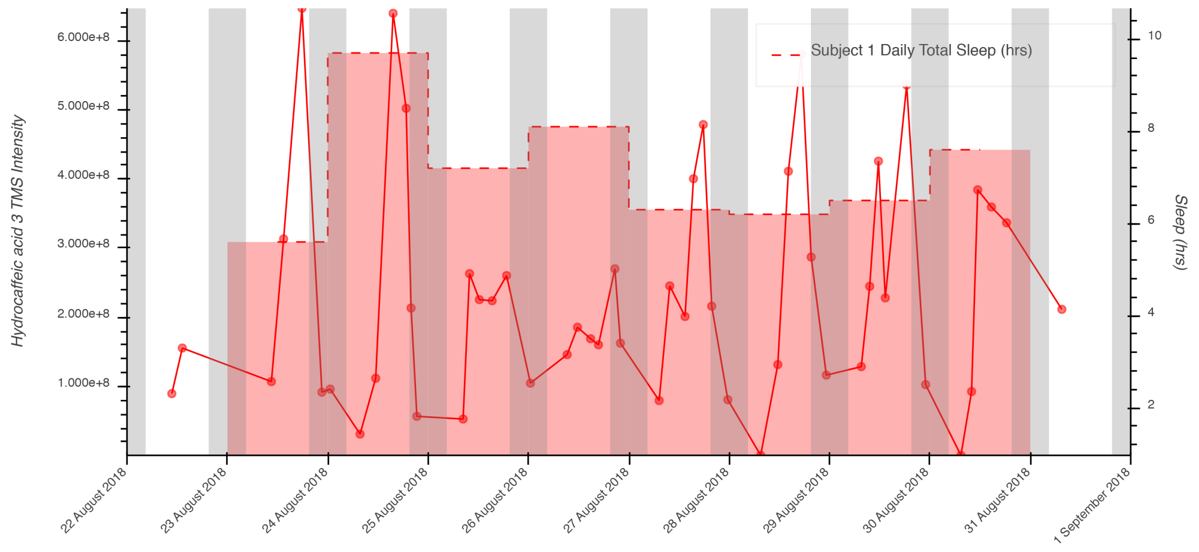

Supplementary Figure 7. Intensity of hydrocaffeic acid 3 TMS over time for both subjects. Sleep (hrs) vs. log2-hydrocaffeic acid 3 TMS (daily average intensity); Spearman's Rho: -0.857, p = 0.0137, q = 0.551, n = 8.

Supplementary Table 1. Summary statistics for biometric measurements

**Daily Mean ± (StDev)**	**Subject 1**	**Subject 2**	**App/Hardware**
Dietary Calories (kcal)	2,904 ± (516)	1,793 ± (448)	Lose It!
Carbohydrates (g)	295 ± (60)	146 ± (37)	Lose It!
Fiber (g)	30 ± (15)	32 ± (11)	Lose It!
Sugar (g)	75 ± (33)	83 ± (37)	Lose It!
Total Fat (g)	120 ± (29)	63 ± (25)	Lose It!
Saturated Fat (g)	46 ± (17)	26 ± (12)	Lose It!
Protein (g)	143 ± (38)	66 ± (27)	Lose It!
Cholesterol (mg)	352 ± (214)	180 ± (112)	Lose It!
Sodium (mg)	3,372 ± (983)	2,063 ± (790)	Lose It!
Active Calories (kcal)	916 ± (214)	NA	Apple Watch
Sleep (hrs)	7.1 (1.2)	NA	Sleep Cycle

Supplementary Dataset 1. Excel file with annotated metabolites and associated diseases from HMDB (hmdb_info.xlsx).

Supplementary Dataset 2. Excel file for HMDB disease associations for all available urine metabolites. (hmdb_disease_metabolite_count.xslx).

Supplementary Dataset 3. Excel file with combined data for metabolites and samples (combined_sample_data.xlsx).

Supplementary Dataset 4. Excel file with correlation analysis results (biometric_correlations.xlsx).
